# Perlecan Facilitates Neuronal Nitric Oxide Synthase Delocalization in Denervation-Induced Muscle Atrophy

**DOI:** 10.3390/cells9112524

**Published:** 2020-11-23

**Authors:** Satoshi Nakada, Yuri Yamashita, Shuichi Machida, Yuko Miyagoe-Suzuki, Eri Arikawa-Hirasawa

**Affiliations:** 1 Japanese Center for Research on Women in Sport, Juntendo University Graduate School of Health and Sports Science, Chiba 270-1695, Japan; s-nakada@juntendo.ac.jp (S.N.); machidas@juntendo.ac.jp (S.M.); 2 Research Institute for Diseases of Old Age, Juntendo University Graduate School of Medicine, Tokyo 113-8421, Japan; yuriyama@juntendo.ac.jp; 3 Department of Molecular Therapy, National Institute of Neuroscience, National Center of Neurology and Psychiatry, Tokyo 187-8502, Japan; miyagoe@ncnp.go.jp

**Keywords:** perlecan, neuronal nitric oxide synthase delocalization, denervation-induced muscle atrophy

## Abstract

Perlecan is an extracellular matrix molecule anchored to the sarcolemma by a dystrophin–glycoprotein complex. Perlecan-deficient mice are tolerant to muscle atrophy, suggesting that perlecan negatively regulates mechanical stress-dependent skeletal muscle mass. Delocalization of neuronal nitric oxide synthase (nNOS) from the sarcolemma to the cytosol triggers protein degradation, thereby initiating skeletal muscle atrophy. We hypothesized that perlecan regulates nNOS delocalization and activates protein degradation during this process. To determine the role of perlecan in nNOS-mediated mechanotransduction, we used sciatic nerve transection as a denervation model of gastrocnemius muscles. Gastrocnemius muscle atrophy was significantly lower in perinatal lethality-rescued perlecan-knockout (*Hspg2*^−/−^-Tg) mice than controls (WT-Tg) on days 4 and 14 following surgery. Immunofluorescence microscopy showed that cell membrane nNOS expression was reduced by denervation in WT-Tg mice, with marginal effects in *Hspg2*^−/−^-Tg mice. Moreover, levels of atrophy-related proteins—i.e., FoxO1a, FoxO3a, atrogin-1, and Lys48-polyubiquitinated proteins—increased in the denervated muscles of WT-Tg mice but not in *Hspg2*^−/−^-Tg mice. These findings suggest that during denervation, perlecan promotes nNOS delocalization from the membrane and stimulates protein degradation and muscle atrophy by activating FoxO signaling and the ubiquitin–proteasome system.

## 1. Introduction

Skeletal muscles are highly plastic and change their mass and properties depending on mechanical stress levels [1,2]. Therefore, the sensing and transmission of mechanical stress are extremely important for regulating cell differentiation, growth, and muscle mass. Links among the proteins of the extracellular matrix, transmembrane, sub-sarcolemma, and cytoskeleton are important for ensuring skeletal muscle integrity [3,4] and for sensing and transmitting mechanical stress (i.e., mechanotransduction) [5,6,7,8]. Mechanical stress reduction, which occurs with hindlimb suspension, denervation, bed rest, and spaceflight, leads to a decrease in the muscle fiber cross-sectional area and atrophy [9,10]. Muscle atrophy during mechanical stress reduction is caused by decreased protein synthesis and increased protein degradation [9,10]. Recent studies have emphasized the importance of ubiquitin–proteasome in triggering atrophy [11,12,13]. The expression of ubiquitin ligases is initiated via FoxO signaling activation during unloading [11,14,15].

Suzuki et al. revealed that mechanical stress reduction is accelerated in response to skeletal muscle atrophy due to the delocalization of neuronal nitric oxide synthase (nNOS) from the sarcolemma to the cytosol [16]. In skeletal muscles, nNOS is a key signaling protein in the dystrophin–glycoprotein complex and is anchored to dystrophin and syntrophin [17,18,19,20,21]. Mechanical stress reduction in the soleus muscle for two weeks results in the delocalization of nNOS to the cytosol. Cytosolic nNOS produces nitric oxide (NO), leading to FoxO signaling activation, which, in turn, stimulates ubiquitin ligases and, thereby, atrophy [16]. Delocalization of nNOS from the dystrophin–glycoprotein complex (DGC), as observed in some muscular disorders such as Duchenne muscular dystrophy, also contributes to chronic pathological wasting [22,23,24]. Additionally, under normal loading conditions, nNOS localizes under the sarcolemma, produces NO in levels proportional to the increase in mechanical stress, and activates satellite cells [25], capillary perfusion [26], and mTOR signaling [27]. These findings indicate that nNOS localization changes according to mechanical stress levels and exerts varying effects depending on its localization. Therefore, although a comprehensive understanding of the mechanism underlying nNOS localization is important, it remains largely unknown.

Some DGC-related proteins have been reported to be involved in this process [6,18,23,28,29,30,31]. Perlecan is a heparan sulfate proteoglycan related to DGC that functions by binding α-dystroglycan [32,33,34]. Previously, we established a perlecan conditional knockout (CKO) mouse model that was perlecan-deficient in all tissues except cartilages; abnormal spontaneous muscle fiber discharges were observed on the needle electromyogram, similar to the human disease (Schwartz–Jampel syndrome, OMIM #255800). This mouse model is useful to study the role of perlecan in skeletal muscles, as well as for studying human diseases. Using this CKO mouse model, we reported that perlecan is associated with skeletal muscle hypertrophy and atrophy, as well as the composition of the muscle fibers [35,36,37]. Thus, we postulated that perlecan might be involved in nNOS delocalization during mechanical stress reduction and could activate protein degradation signals and induce muscle atrophy. We hypothesized that perlecan facilitates nNOS delocalization during the early stages of mechanical unloading and that this delocalization is linked to FoxO signaling activation, protein degradation, and muscle atrophy. These processes were investigated in the present study.

## 2. Materials and Methods

### 2.1. Animals

Perinatal lethality-rescued perlecan-knockout (*Hspg2*^−/−^-Tg) and control (WT-Tg) mice were used in this study [35,38]. Typically, perlecan-deficient mice die in the perinatal period due to defective cartilage development [39,40]. Therefore, we previously established a perlecan transgenic mouse line (WT-Tg and *Hspg2*^−/−^-Tg) in which cartilage abnormalities were repaired by expressing recombinant perlecan in cartilage using the cartilage-specific *COL2A1* promoter/enhancer [41]. WT-Tg and *Hspg2*^−/−^-Tg mice were maintained in the C57BL/6 background. The study used 17 female mice, aged 12–14 weeks, in each group. In each group, *n* = 11 was used to observe acute biochemical changes after denervation (Western blotting, *n* = 6; immunofluorescence, *n* = 5), and *n* = 6 was used to observe long-term morphological changes. The mice were housed in an animal facility with regulated temperature (22 °C), humidity (60%), and light–dark cycle (12 h light and 12 h dark). All animal protocols were approved by the Animal Care and Use Committee of Juntendo University (identification code: 280190). The mice were genotyped by PCR, using DNA extracted from tail biopsies. The wild type (WT) allele was detected by the primer pair WT-F and WT/*Hspg2*^−^-R, the *Hspg2*^−^ allele was detected by the primer pair *Hspg2*^−^-F and WT/*Hspg2*^−^-R, and the recombinant perlecan transgenic allele was detected by the primer pair Tg-F and Tg-R. The sequences of the primer used were as follows: WT-F, CCCGAGTCTCTGTCAACGCACCC; *Hspg2*^−^-F, CGCCTTCTATCGCCTTCTTGACGAGTTC; WT/*Hspg2*^−^-R, CACAGCGCCACAACTTGAGAGCACAG; Tg-F, CCCAGGTGGAGGGCCGTACT; Tg-R, GCCGCCAGAACTCCTCTCG.

### 2.2. Denervation Surgery

The mice were subjected to unilateral denervation surgery of the gastrocnemius muscle under anesthesia (sodium pentobarbital, 60 mg/kg body weight, intraperitoneal injection). An incision was made in the skin of the left thigh, and the thigh muscles were separated to expose the sciatic nerve. A small piece of nerve (5 mm) was resected, and the skin was closed with a 5-0 nylon suture (denervated side (DEN)). A sham operation was performed in the right thigh of each animal as an internal control (control side (CON)).

### 2.3. Muscle Sampling

On day 4 or 14 following surgery, the mice were weighed and sacrificed via cervical dislocation. The gastrocnemius muscles were immediately extracted and weighed. The muscle samples obtained on day 4 were used for biochemical and immunofluorescence analyses. Previous studies have shown that activation of protein degradation signals and delocalization of sarcolemma nNOS occur in 3–4 days of denervation-induced muscle atrophy [16,42]. For biochemical evaluation, each muscle was quickly frozen, ground into powder in liquid nitrogen, and stored at −80 °C until analysis. For immunofluorescence evaluation, each muscle was covered with an embedding medium (Tissue-Tek OCT compound; Sakura Finetechnical, Tokyo, Japan), snap-frozen in isopentane chilled in liquid nitrogen, and stored at −80 °C until analysis.

### 2.4. Immunofluorescence Microscopy

Frozen samples embedded in OCT compound were sliced using a cryostat to obtain 10-µm-thick transverse sections. These sections were attached to glass slides and fixed in ice-cold acetone for 10 min, followed by blocking with 2% bovine serum albumin for 1 h. The sections were then reacted with primary antibodies (anti-nNOS antibody 1:100, #61-7000, Thermo Fisher Scientific, Waltham, MA, USA; anti-dystrophin antibody 1:100, VPD505; Vector Laboratories, Burlingame, CA, USA) at 4 °C overnight. After incubation, sections were re-incubated with the fluorescence-conjugated secondary antibodies (Alexa Fluor 488 conjugated goat anti-rabbit IgG 1:400 and Alexa Fluor 546 conjugated goat anti-mouse IgG 1:400, Thermo Fisher Scientific) for 1 h at room temperature. Subsequently, glass coverslips were mounted to the sections using an aqueous mounting medium (Vectashield antifade mounting medium, Vector Laboratories). Cross-sectional images were captured following identical exposure times to optimize the comparison using a digital fluorescence microscope (BZX-700; Keyence, Tokyo, Japan). Tiled images of whole muscle sections were captured as 10-µm-thick z-stacks with a 0.4 µm step size. All images obtained by tile scans were used for analysis, and the entire section was analyzed. For direct comparisons, samples from the four experimental treatments (WT-Tg, *Hspg2*^−/−^-Tg × CON, DEN) were treated under the same conditions. The fluorescence intensities in the captured images were measured using ImageJ software [43]. To detect the membrane nNOS expression, the regions of nNOS staining overlapping with dystrophin immunoreactivity-positive area were extracted using ImageJ. The fluorescence intensity of membrane nNOS was evaluated, and the mean and frequency distribution of the gray value was determined by assessing the number of pixels of each gray value obtained from the whole muscle section.

### 2.5. Total Protein Extraction

Powdered, frozen samples were weighed and homogenized using a hand-held polytron homogenizer (Ultra-Turrax T8; IKA Labortechnik, Staufen, Germany) in ice-cold RIPA buffer containing 25 mM Tris-HCl (pH 7.4), 1% NP-40, 0.1% sodium dodecyl sulfate (SDS), 1% sodium deoxycholate, 5 mM EDTA, 150 mM NaCl, protease inhibitor cocktail (Complete Mini; Roche, Basel, Switzerland), and phosphatase inhibitor cocktail (PhosSTOP; Roche). Homogenates were centrifuged at 10,000× *g* for 15 min at 4 °C, and supernatants were collected and used for Western blot analyses.

### 2.6. Western Blotting

The levels of nNOS, FoxO1a, FoxO3a, atrogin-1, and Lys48-polyubiquitinated protein were measured in the total protein samples. Protein concentrations in the extracts were determined using a protein quantification kit (Pierce BCA Protein Assay Kit; Thermo Fisher Scientific), and samples were diluted to the same concentration using an appropriate homogenization buffer. Samples containing 24 µg of total protein were separated by SDS-PAGE and transferred to polyvinylidene difluoride membranes. After transfer, the membranes were stained with Ponceau S and scanned under a white light with a chemiluminescence detector (Amersham Imager 600; GE Healthcare, Little Chalfont, UK). Images were used to confirm equal loading in all lanes [44]. After scanning, the membranes were destained with Tris-buffered saline containing 0.1% Tween-20 and blocked with 5% skimmed milk in the same buffer for 1 h at room temperature. The membranes were then washed and incubated overnight with primary antibodies at 4 °C, followed by incubation with horseradish peroxidase-conjugated anti-rabbit (#7074S, Cell Signaling Technology, Danvers, MA, USA) or anti-mouse antibodies (#7076S, Cell Signaling Technology) for 1 h at room temperature. Immunodetection was conducted using chemiluminescent reagents (Luminata Forte; Millipore, Billerica, MA, USA) and a chemiluminescence detector (Amersham Imager 600). The band intensities in the captured images were measured using ImageJ software (NIH, Bethesda, MD, USA) [43]. For direct comparisons, samples from the four experimental treatments (WT-Tg, *Hspg2*^−/−^-Tg × CON, DEN) were resolved on the same gel. The following primary antibodies were used: Anti-nNOS antibody (1:1000, #61-7000, Thermo Fisher Scientific), anti-FoxO1a antibody (1:1000, #2880, Cell Signaling Technology), anti-FoxO3a antibody (1:1000, #12829 Cell Signaling Technology), anti-atrogin-1 antibody (1:1000, ab168372, Abcam, Cambridge, UK), and anti-ubiquitin, Lys48-Specific antibody (1:1000, 05-1307, Millipore).

### 2.7. Statistical Analyses

Data are presented as the mean ± SD. Graphpad Prism (Graphpad Software, San Diego, CA, USA) was used for the statistical analyses. The results of the frequency distribution of nNOS immunofluorescence were analyzed by two-way analysis of variance (surgery condition × gray value). Following significant main or interaction effects, Sidak post-hoc tests were used for pairwise comparisons between surgery conditions. Data were also analyzed for the effect of denervation in each group using a paired *t*-test (CON vs. DEN). For between-group comparisons, the percent change from CON to DEN for each group was calculated and compared using unpaired *t*-tests. Results with *p* < 0.05 were considered statistically significant.

## 3. Results

### 3.1. Changes in Muscle Weight after Denervation Surgery

**Figure 1:**
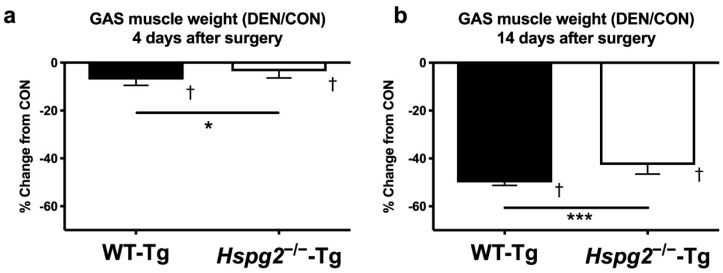
Percent change in gastrocnemius (GAS) muscle weight 4 days (**a**) and 14 days (**b**) after denervation surgery. To examine the effect of denervation on the gastrocnemius muscle, the sciatic nerve in the left hind limb was excised (denervated side (DEN)), and the right hind limb was subjected to a sham operation (control side (CON)). The wet weight of the gastrocnemius muscle was measured on days 4 (**a**) and 14 (**b**) following surgery. The effect of denervation on muscle weight was determined by comparing DEN with CON. In each group at each time point, the DEN muscle weight was significantly lower than the CON muscle weight. At each time point, the percent change from CON to DEN samples was significantly different between groups. †, significant differences between DEN and CON within the same group (*p* < 0.05, *n* = 6). * and ***, the percent change (DEN/CON) was significantly different between the WT-Tg and *Hspg2*^−/−^-Tg mice (* *p* < 0.05, *** *p* < 0.001, *n* = 6). Values are expressed as the mean ± SD.

The relative weights of the gastrocnemius muscles (muscle weight/body weight) were measured on days 4 and 14 following denervation surgery. The relative weight of the control side (CON) of the gastrocnemius muscle was not significantly different between the WT-Tg and *Hspg2*^−/−^-Tg mice on days 4 and 14 (day 4: WT-Tg, 4.93 ± 0.18 mg/g body weight (BW) and *Hspg2*^−/−^-Tg, 4.94 ± 0.16 mg/g BW, *p* = 0.99; day 14: WT-Tg, 4.97 ± 0.11 mg/g BW and *Hspg2*^−/−^-Tg, 5.06 ± 0.34 mg/g BW, *p* = 0.73). In both groups, the relative weight of the gastrocnemius muscle of the denervated side (DEN) was significantly lower than that of the CON side (day 4: WT-Tg, *p* = 0.0004 and *Hspg2*^−/−^-Tg, *p* = 0.0148; day 14: WT-Tg, *p* < 0.0001 and *Hspg2*^−/−^-Tg, *p* < 0.0001). Comparing the percent change from CON to DEN between the groups, the *Hspg2*^−/−^-Tg mice showed a significantly smaller decrease than the WT-Tg mice at both time points (day 4: WT-Tg, 7.0 ± 2.5% decrease and *Hspg2*^−/−^-Tg, 3.6 ± 2.8% decrease, *p* = 0.030 (Figure 1a); day 14: WT-Tg, 50.1 ± 1.2% decrease and *Hspg2*^−/−^-Tg, 42.7 ± 3.9% decrease, *p* = 0.004 (Figure 1b)). These results suggest that the *Hspg2*^−/−^-Tg mice were more tolerant to denervation-induced muscle atrophy than the WT-Tg mice.

### 3.2. Changes in Membrane nNOS Expression

**Figure 2:**
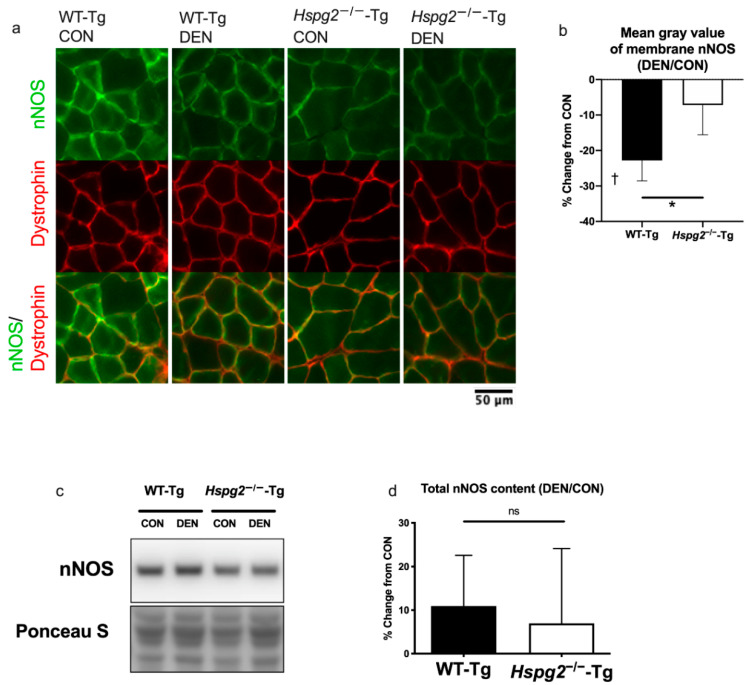
Changes in membrane neuronal nitric oxide synthase (nNOS) expression 4 days after denervation surgery. (**a**) nNOS and dystrophin co-localized 4 days after denervation surgery. Immunofluorescence images of the gastrocnemius muscles showing individual as well as co-localized expression of nNOS and dystrophin. The fluorescence intensity of membrane nNOS decreased after denervation in the WT-Tg mice, whereas this effect was lower in the *Hspg2*^−/−^-Tg mice. (**b**) Percent change (from CON to DEN) of the mean gray value of membrane nNOS. Significant differences were observed between the WT-Tg CON and DEN samples (*p* < 0.05), whereas no significant differences were noted between the *Hspg2*^−/−^-Tg CON and DEN samples (*p* = 0.42). The percent changes in the mean gray value of membrane nNOS between the DEN and CON muscles were significantly different in the WT-Tg mice compared to the *Hspg2*^−/−^-Tg mice. Values are expressed as the mean ± SD. †, DEN was significantly different from CON within the same group (*p* < 0.05, *n* = 5). *, significant differences between the WT-Tg and *Hspg2*^−/−^-Tg mice (*p* < 0.05, *n* = 5). (**c**) Western blotting results represent the levels of total nNOS and total protein by Ponceau S staining. (**d**) Percent change (from CON to DEN) in the total nNOS protein content. The total nNOS content did not significantly change between the CON muscle and the DEN muscle in either the WT-Tg or the *Hspg2*^−/−^-Tg mice. Percent change in the total nNOS did not differ between the WT-Tg and *Hspg2*^−/−^-Tg mice.

Changes in the expression of cell membrane nNOS were observed by fluorescence immunostaining. The typical expression patterns of nNOS and dystrophin are shown in Figure 2a. The expression pattern of dystrophin, one of the membrane-related proteins, was comparable among the four groups. On the contrary, the nNOS expression pattern was different under the four conditions. In the WT-Tg CON samples, nNOS was strongly expressed on the cell membrane, whereas in the WT-Tg DEN samples, membrane nNOS expression was reduced. In contrast, there was no difference in membrane nNOS expression between CON and DEN in the *Hspg2*^−/−^-Tg mice.

The mean gray value of membrane nNOS significantly differed between the CON and DEN samples in the WT-Tg mice (Figure 2b, *p* < 0.05). In contrast, there was no difference in the mean gray value between the CON and DEN samples in the *Hspg2*^−/−^-Tg mice (*p* = 0.42). The mean gray value of the muscle of the *Hspg2*^−/−^-Tg CON sample tended to decrease compared to that of the WT-Tg CON sample, but there was not a significant difference (WT-Tg, 54.7 ± 14.48; *Hspg2*^−/−^-Tg, 35.28 ± 13.91; *p* = 0.075). Additional analysis corrected by the ratio of the mean gray value of nNOS to the mean gray value of dystrophin revealed that the expression level of membrane nNOS was significantly lower in the *Hspg2*^−/−^-Tg CON sample compared to the WT-Tg CON sample (WT-Tg, 2.05 ± 0.21; *Hspg2*^−/−^-Tg, 1.24 ± 0.45; *p* = 0.0017). The rate of change in the mean gray value from CON to DEN was significantly different in the WT-Tg compared to the *Hspg2*^−/−^-Tg mice (*p* < 0.05). These results indicate that membrane nNOS expression was decreased after denervation in the WT-Tg mice; however, less changes were observed in the *Hspg2*^−/−^-Tg mice. Dystrophin immunostaining was also performed on the same sections, which confirmed that the results of the nNOS expression changes were accurate (Figure S1).

Additionally, Western blotting was performed on the total protein samples to confirm that the whole muscle nNOS level did not change in the WT-Tg and *Hspg2*^−/−^-Tg mice (Figure 2c,d). After four days of denervation, there was no significant difference in the total nNOS protein content of the CON muscle between the WT-Tg and *Hspg2*^−/−^-Tg mice (WT-Tg, 1.00 ± 0.15 A.U.; *Hspg2*^−/−^-Tg, 0.82 ± 0.12 A.U.; *p* = 0.11). In addition, no significant changes were observed in the total nNOS protein content in the CON and DEN muscles of both groups (WT-Tg, *p* = 0.07; *Hspg2*^−/−^-Tg, *p* = 0.37). Moreover, no significant differences were observed in the rate of change from CON to DEN between the WT-Tg and *Hspg2*^−/−^-Tg mice (*p* = 0.65).

Since no changes in nNOS protein levels were observed in the WT-Tg mice, and yet the membrane nNOS expression decreased, this suggests that nNOS delocalized from the membrane to the cytosol as a result of denervation in the WT-Tg mice. However, these denervation-associated changes in nNOS localization were suppressed in the *Hspg2*^−/−^-Tg mice.

### 3.3. FoxO Signaling and Ubiquitin—Proteasome Signaling Activation

**Figure 3:**
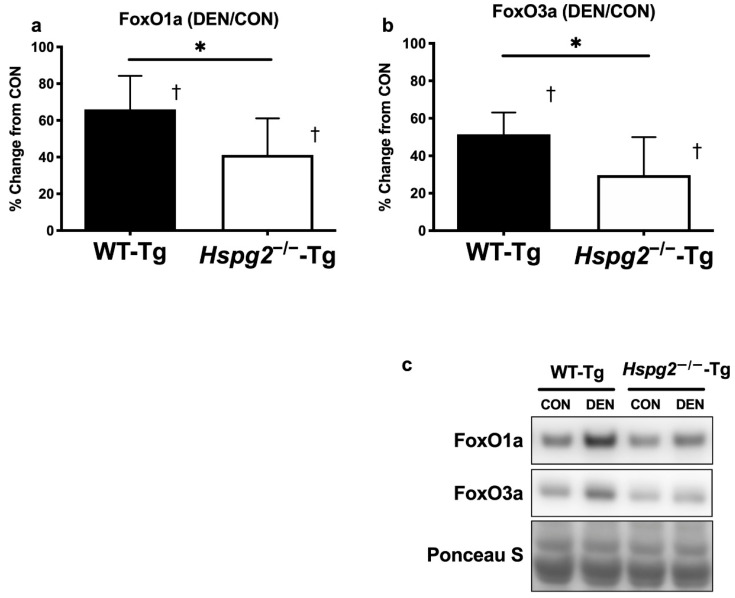
Changes in FoxO protein expression 4 days after denervation surgery. FoxO protein content in the gastrocnemius muscle at 4 days after surgery was measured by Western blotting. Percent changes in FoxO1a (**a**) and FoxO3a (**b**) protein contents were determined by comparing the DEN (denervated) and CON (control) muscles. In the WT-Tg and *Hspg2*^−/−^-Tg mice, FoxO1a and FoxO3a protein expression increased significantly in the DEN compared to in the CON samples. The percent changes in FoxO1a and FoxO3a protein content between DEN and CON were significantly different between the WT-Tg and *Hspg2*^−/−^-Tg mice. †, DEN significantly differed from CON within the same group (*p* < 0.05, *n* = 6). *, significant difference between the two groups (* *p* < 0.05, *n* = 6). Values are expressed as the mean ± SD. (**c**) The Western blotting results demonstrate the levels of FoxO1a, FoxO3a, and total protein by Ponceau S staining.

During skeletal muscle atrophy, proteins are degraded by ubiquitin–proteasome signaling, which is activated by the FoxO signaling pathway [11,12,45]. To determine the effect of perlecan on FoxO activation, we measured the protein levels of FoxO1a and FoxO3a in both the CON and DEN gastrocnemius muscles (Figure 3). Four days after denervation, the total amount of FoxO1a protein of the control side (CON) of the gastrocnemius muscle was not significantly different between the WT-Tg and *Hspg2*^−/−^-Tg mice (WT-Tg, 1.00 ± 0.18 A.U.; *Hspg2*^−/−^-Tg, 1.15 ± 0.26 A.U.; *p* = 0.45). In both groups, the total amount of FoxO1a protein was significantly higher in the DEN muscle than in the CON muscle (WT-Tg, *p* = 0.0003; *Hspg2*^−/−^-Tg, *p* = 0.0039). However, the percent change in FoxO1a (CON to DEN) in the *Hspg2*^−/−^-Tg mice was significantly lower than in the WT-Tg mice (*p* = 0.049). Similarly, the total FoxO3a protein content of the CON muscle was not significantly different between the WT-Tg CON and *Hspg2*^−/−^-Tg CON mice (WT-Tg, 1.00 ± 0.47 A.U.; *Hspg2*^−/−^-Tg, 1.07 ± 0.51 A.U.; *p* = 0.97). In both groups, the total amount of FoxO3a protein was significantly higher in the DEN muscle than in the CON muscle (WT-Tg, *p* = 0.0001; *Hspg2*^−/−^-Tg, *p* = 0.0157). The percent change in FoxO3a content (CON to DEN) in the *Hspg2*^−/−^-Tg mice was significantly lower than in the WT-Tg mice (*p* = 0.046).

**Figure 4:**
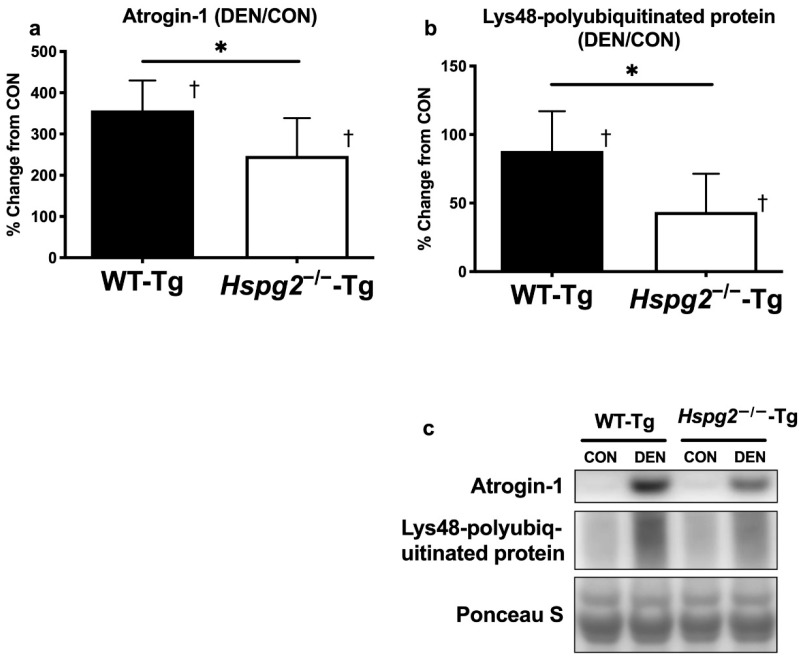
Changes in ubiquitin ligase and polyubiquitinated protein content 4 days after denervation surgery. Atrogin-1 and Lys48–polyubiquitin protein contents in the gastrocnemius muscle were measured 4 days after surgery by performing total protein extraction and Western blotting. Percent changes in atrogin-1 (**a**) and Lys48–polyubiquitin (**b**) protein contents were determined by comparing the DEN and CON muscles. In the WT-Tg and *Hspg2*^−/−^-Tg mice, atrogin-1 and Lys48–polyubiquitin protein expression in the DEN samples increased significantly compared to that in the CON samples. For atrogin-1 and Lys48–polyubiquitin protein content, the percent changes between the DEN and CON muscles were significantly different between the WT-Tg and *Hspg2*^−/−^-Tg mice. †, DEN was significantly different from CON within the same group (*p* < 0.05, *n* = 6). *, significantly different between the two groups (* *p* < 0.05, *n* = 6). Values are the mean ± SD. (**c**) Western blotting results represent the levels of atrogin-1, Lys48–polyubiquitin protein, and total protein by Ponceau S staining.

Furthermore, we investigated the effect of perlecan on the activation of the ubiquitin–proteasome signaling ubiquitin E3 ligase, atrogin-1. Ponceau S staining of the polyvinylidene difluoride membranes confirmed equal loading of total protein into each lane (Figure 4a,c). The total atrogin-1 protein content was not significantly different between the CON muscles in either group (WT-Tg, 1.00 ± 0.68 A.U.; *Hspg2*^−/−^-Tg, 1.28 ± 0.67 A.U.; *p* = 0.91) and was significantly higher in the DEN muscle than in the CON in both groups (WT-Tg, *p* < 0.0001; *Hspg2*^−/−^-Tg, *p* = 0.0012). The percent change in atrogin-1 content (CON to DEN) in the *Hspg2*^−/−^-Tg mice was significantly lower than in the WT-Tg mice (*p* = 0.043).

We observed that the total protein content of Lys48–polyubiquitin chains was not significantly different between the CON muscles in either group (WT-Tg, 1.00 ± 0.20 A.U.; *Hspg2*^−/−^-Tg, 1.25 ± 0.31 A.U.; *p* = 0.27) and was significantly higher in the DEN muscle than in the CON muscle in both groups (WT-Tg, *p* = 0.0024; *Hspg2*^−/−^-Tg, *p* = 0.025; Figure 4b). Moreover, the percent change (CON to DEN) in the *Hspg2*^−/−^-Tg mice was significantly lower than in the WT-Tg (*p* = 0.038). These results suggest that the activation of FoxO signaling and the ubiquitin–proteasome pathway was inhibited in the *Hspg2*^−/−^-Tg mice during denervation-induced atrophy.

## 4. Discussion

This study demonstrated that a deficiency of perlecan, a heparan sulfate proteoglycan, attenuates denervation-induced skeletal muscle atrophy. In addition, perlecan deficiency suppresses the delocalization of nNOS from the sarcolemma. Furthermore, denervated perlecan-deficient muscles also exhibit decreased FoxO signaling and ubiquitin–proteasome system activation, which are triggered by nNOS delocalization.

### 4.1. Perlecan Promotes Skeletal Muscle Atrophy Following Denervation

Perlecan is a proteoglycan comprising a heparan sulfate chain and core proteins. It is an extracellular matrix protein that interacts with various molecules [46,47]. In skeletal muscles, perlecan has been reported to be involved in multiple functions [48], such as AChE localization in the post-synaptic membrane [49,50], myoblast adhesion to collagen IV [51], and histone 1 binding to the plasma membrane [52]. Our previous studies indicated that perlecan affects skeletal muscle hypertrophy, atrophy [35,36], and fiber-type composition [37]. Our study on skeletal muscle hypertrophy and myostatin signaling revealed that the expression of myostatin and its type I receptor was substantially decreased in overloaded perlecan-deficient muscles compared to muscles from control mice [35]. The objective of the present study was to investigate the effect of perlecan on mechanotransduction changes during skeletal muscle atrophy. Perlecan deficiency causes hyperexcitability of skeletal muscles by inhibiting AChE degradation at neuromuscular junctions [50] and by persistent axonal depolarization of the motor nerve [53,54,55]. Moreover, as electrical stimulation of skeletal muscles is known to suppress muscle atrophy [56], the hyperexcitability of skeletal muscles might also mask mechanotransduction changes. Thus, to eliminate the effect of motor neuron and neuromuscular junction changes and to observe only the effects of perlecan deficiency on mechanotransduction during skeletal muscle atrophy, sciatic nerve resection was performed.

Gastrocnemius muscle obtained four days following surgery was evaluated for biochemical changes during the early stages of atrophy. It has been reported that the activation of protein degradation signals and the delocalization of sarcolemma nNOS occur at this time point during denervation-induced muscle atrophy [16,42]. Gastrocnemius muscle was also obtained at 14 days after surgery to examine long-term morphological changes. Since there was no significant difference between the wild-type and perlecan-deficient gastrocnemius muscle weight of the control legs, we speculate that perlecan deficiency does not affect the muscle mass of the innervated skeletal muscles. Both wild-type and perlecan-deficient gastrocnemius muscles exhibited atrophy following denervation (Figure 1). Wild-type gastrocnemius muscle showed a 7.0% and 50.1% decrease on days 4 and 14 following denervation, respectively. In contrast, the rate of muscle atrophy was lower in perlecan-deficient gastrocnemius muscle than in wild-type gastrocnemius muscle at both time points (3.6% and 42.7% decrease on days 4 and 14, respectively). These results indicate that perlecan-deficient gastrocnemius muscle is more resistant to muscle atrophy than wild-type gastrocnemius muscle. Xu et al. reported that perlecan-deficient plantaris muscle shows less muscle atrophy due to tenotomy than wild-type plantaris muscle [35]. These results suggest that perlecan inhibits the muscle atrophy induced by mechanical stress reductions, irrespective of the differences between muscle atrophy models or differences in target muscles.

### 4.2. Perlecan Facilitates nNOS Delocalization, FoxO Signaling, and Ubiquitin—Proteasome System Activation

Previous studies have established that nNOS delocalizes from the sarcolemma to the cytosol during skeletal muscle atrophy or in certain muscular disorders [16,22,23,24,57,58,59]. Delocalization of nNOS activates the FoxO signaling pathway, which promotes muscle atrophy and contributes to chronic pathological wasting [60]. Although the mechanisms underlying nNOS delocalization are not fully understood, mechanical stress reduction and DGC-related proteins are reportedly involved in this process [6,16,18,23,28,29,30,31,57,58,59].

Delocalization of nNOS from the sarcolemma has been observed in muscle atrophy studies involving hind limb suspension and denervation of rodents [16,57,58,59]. Therefore, it has been suggested that nNOS delocalization is induced by mechanical stress reduction in skeletal muscles. The mechanism by which mechanical stress reduction causes nNOS delocalization is unclear but could be explained by conformational changes in the dystrophin nNOS-binding domain. An investigation into the mechanical stability of dystrophin revealed that a central domain containing the nNOS-binding domain of dystrophin changes conformation in response to force [61]. As reported for the integrin–talin–vinculin complex, some mechanosensing molecules change their conformation in response to force and expose the binding site to allow ligand binding [62,63]. Therefore, for dystrophin and nNOS, decreased mechanical stress might weaken the binding ability of nNOS associated with dystrophin conformation leading to nNOS delocalization.

DGC-related proteins are reported to be involved in nNOS delocalization. As dystrophin and syntrophin each have an nNOS-binding site and are directly involved in nNOS localization to the sarcolemma, their deficiency might lead to nNOS delocalization [6,18]. Furthermore, although sarcoglycans and biglycans are not directly bound to nNOS, their deficiency is associated with nNOS delocalization [23,28,29,30,31]. Collectively, these findings indicate that the binding of nNOS to the sarcolemma is influenced by various DGC-related proteins.

This study observed the effect of DGC-related proteoglycan and perlecan on nNOS delocalization following denervation. Our immunofluorescence microscopy results showed that the nNOS expression in the membrane of wild-type gastrocnemius muscle decreased following denervation. In contrast, decreased nNOS expression in the membrane was suppressed in perlecan-deficient muscle. Moreover, the Western blotting results showed that total nNOS protein in wild-type muscle did not significantly decrease after denervation. This suggests that the delocalization of nNOS from the sarcolemma to the cytosol was induced by denervation, consistent with the results of tail suspension performed in previous studies [16,57,58,59]. Additionally, the expression level of nNOS in both the membrane and total muscle did not change after denervation in the *Hspg2*^−/−^-Tg mice. Thus, it was considered that perlecan promotes the delocalization of nNOS from the sarcolemma to the cytosol during mechanical stress reduction.

The mechanism by which perlecan facilitates nNOS delocalization during mechanical stress reduction might be explained by the effect of perlecan on basement membrane stiffness. Recent studies based on *Drosophila* spp. have revealed that perlecan attenuates basement membrane stiffness [64,65,66,67]. Therefore, perlecan might also reduce the stiffness of the basement membrane in vertebrate skeletal muscles. The decrease in basement membrane stiffness reduces the transmission of mechanical stress, facilitating nNOS delocalization during mechanical stress reduction. Furthermore, perlecan is a multifunctional molecule that binds to several other molecules, including growth factors, extracellular molecules, cell surface receptors such as integrin, and dystroglycans [68]. These interactions with diverse molecules may affect the response of muscles to mechanical stress.

Previous studies have reported that delocalized nNOS activates FoxO signaling and the ubiquitin–proteasome system [16,57,58,59]. Therefore, we examined FoxO signaling and the ubiquitin–proteasome system. Significant increases in FoxO1a and FoxO3a protein contents were observed in wild-type gastrocnemius muscle following denervation (Figure 3a,b). In contrast, the increase in both FoxO proteins following denervation was suppressed in perlecan-deficient gastrocnemius muscles (FoxO1a and FoxO3a were suppressed by 37.6% and 42.3%, respectively, compared to the rate of increase in wild-type muscle). In addition, nNOS overexpression in myotubes increases FoxO3a protein expression [16]. Therefore, the increase in FoxO1a and FoxO3a protein content following denervation observed in the present study might be explained by the increase in cytosolic nNOS content due to nNOS delocalization, suggesting that perlecan deficiency inhibits these processes. Increases in the contents of FoxO1a and FoxO3a proteins upregulate ubiquitin ligase expression [11,14,15], which labels proteins with polyubiquitin at Lys48 for degradation by proteasomes [12,13]. Therefore, we investigated the ubiquitin ligase content and observed a significant increase in atrogin-1 protein expression in wild-type gastrocnemius muscle following denervation. In contrast, the increase in atrogin-1 expression was suppressed by 30.9% in perlecan-deficient gastrocnemius muscle (Figure 4a). Moreover, the increase in Lys48–polyubiquitin proteins following denervation was enhanced in wild-type gastrocnemius but suppressed by 50.6% in the perlecan-deficient gastrocnemius (Figure 4b). In addition, a comparison of innervated gastrocnemius muscles showed no significant difference in the expression of FoxO1a, FoxO3a, atrogin1, and Lys48–polyubiquitin protein contents between the wild-type muscle and the perlecan-deficient muscle. These results indicate that perlecan deficiency does not affect activation of FoxO signaling or the ubiquitin–proteasome system in innervated muscles.

In summary, the contents of FoxO1a, FoxO3a, atrogin-1, and Lys48–polyubiquitin proteins increased in wild-type skeletal muscles following denervation, but these increases were consistently suppressed in perlecan-deficient skeletal muscles. Activation of the ubiquitin–proteasome system in the skeletal muscles caused a decrease in muscle mass by promoting protein degradation, which is consistent with the muscle weight results in the current study (Figure 1). This suggests that perlecan regulates the activation of FoxO signaling and the ubiquitin–proteasome system in denervation-induced muscle atrophy. Furthermore, cytosolic nNOS reportedly activates FoxO signaling, indicating that nNOS delocalization is one of the mechanisms of perlecan induced FoxO signaling. Further, perlecan deficiency suppressed the activation of protein degradation markers (FoxO1a, 37.6%; FoxO3a, 42.3%; atrogin-1, 30.9%; Lys48–polyubiquitin protein, 50.6%) in denervated muscle, as compared to the increase in denervated wild-type skeletal muscles.

## 5. Conclusions

**Figure 5:**
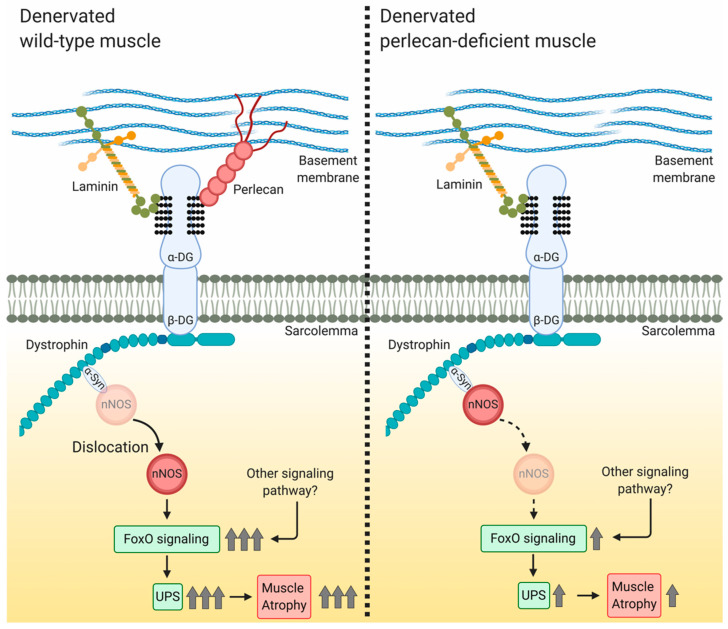
Model showing the involvement of perlecan in nNOS delocalization in denervation-induced muscle atrophy. In denervated wild-type muscle, nNOS delocalizes from the sarcolemma to the cytosol and activates FoxO signaling, after which the ubiquitin–proteasome system facilitates muscle atrophy. In denervated perlecan-deficient muscle, nNOS delocalization inhibits FoxO signaling activation, leading to attenuation of the ubiquitin–proteasome system and inhibition of muscle atrophy. α-DG, α-dystroglycan; β-DG, β-dystroglycan; α-Syn, α-syntrophin; UPS, ubiquitin–proteasome system. Created using BioRender.com.

During skeletal muscle denervation, perlecan promotes nNOS delocalization from the membrane to the cytosol, where delocalized nNOS promotes protein degradation and muscle atrophy by activating FoxO signaling and the ubiquitin–proteasome system (Figure 5). Although this study was limited to muscle atrophy based on denervation, it is the first report to show that perlecan is related to nNOS delocalization and impacts skeletal muscle mechanotransduction. In contrast to other DGC-related molecules, such as sarcoglycans and biglycans [23,28,29,30,31], perlecan deficiency suppresses the delocalization of nNOS, and therefore, it is considered a promoter of nNOS delocalization. Further studies, particularly structural biological analyses of the DGC nNOS-binding domain, will reveal the precise mechanism underlying the effect of perlecan on nNOS delocalization.

## Supplementary Materials

The following are available online at https://www.mdpi.com/2073-4409/9/11/2524/s1, Figure S1, (**a**) Membrane nNOS-extracted images obtained by image processing. Based on the nNOS and dystrophin immunostaining images of the gastrocnemius muscle, the nNOS signal co-localized with dystrophin was extracted as a membrane nNOS by image processing. The right side of the section was the deep region of the muscle. Fluorescence intensity is indicated by pseudo-colors. In the upper right corner, the pseudo-color conversion lookup table is shown. Inserts show magnified views of each image. (**b**) Frequency distribution of gray values for membrane nNOS immunostaining of the WT-Tg gastrocnemius muscle sections. Compared to WT-Tg CON (control) samples, the gray value distribution shifted to lower values in the WT-Tg DEN (denervated) samples. *: a significant difference was observed between CON and DEN. (**c**) Frequency distribution of gray values for membrane nNOS immunostaining of the *Hspg2*^−/−^-Tg gastrocnemius muscle sections. A smaller shift in the frequency distribution was observed compared to *Hspg2*^−/−^-Tg CON and DEN. (**d**) Immunofluorescence images of gastrocnemius muscle whole cross-sections showing dystrophin expression. (**e**) Frequency distribution of gray values for dystrophin immunostaining of the WT-Tg gastrocnemius muscle sections. Gray value distribution did not change significantly between WT-Tg CON and WT-Tg DEN (*p* > 0.05). (**f**) Frequency distribution of gray values for dystrophin immunostaining of the *Hspg2*^−/−^-Tg gastrocnemius muscle sections. Comparison of *Hspg2*^−/−^-Tg CON and DEN revealed significant differences at lower gray values (*p* < 0.05), but the overall shift was slight. (**g**) Percent change (from CON to DEN) in the mean gray value of dystrophin. A significant difference was not observed between WT-Tg CON and DEN nor between *Hspg2*^−/−^-Tg CON and DEN (*p* > 0.05). Values are expressed as the mean ± SD. (**h**) The corrected nNOS gray value calculated from the membrane nNOS gray value and dystrophin immunostaining gray value. †: a significant difference was observed between WT-Tg CON and WT-Tg DEN (*p* < 0.05). *: a significant difference was observed between WT-Tg CON and *Hspg2*^−/−^-Tg CON (*p* < 0.05).
