# Supplementary material for: Perlecan Facilitates Neuronal Nitric Oxide Synthase Delocalization in Denervation-Induced Muscle Atrophy
Source: Cells. 2020 Nov 23;9(11):2524. doi: 10.3390/cells9112524 (PMC7700382; doi:10.3390/cells9112524)
Supplement: Supplementary file 1 [file cells-09-02524-s001.pdf]

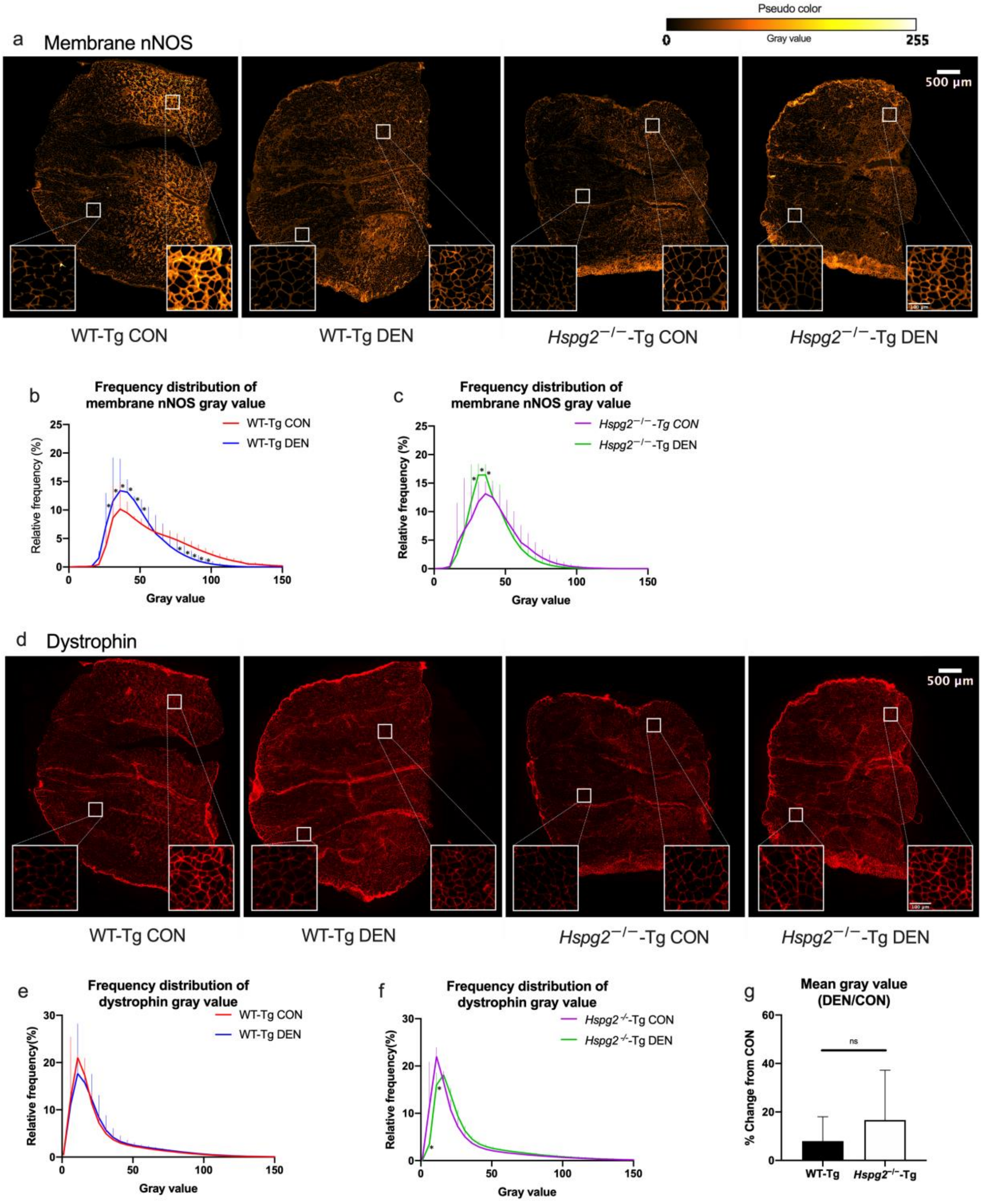

h

The corrected nNOS gray value (Membrane nNOS / dystrophin)			
	WT-Tg		
	Mean	SD	N
CON	2.05	0.21	5
DEN	1.51	0.30	5
	Hspg2$^{-/-}$-Tg		
	Mean	SD	N
CON	1.24	0.45	5
DEN	1.03	0.24	5

† (WT-Tg CON vs DEN); * (WT-Tg CON vs *Hspg2*$^{-/-}$-Tg CON)

Figure S1. (**a**) Membrane nNOS-extracted images obtained by image processing. Based on the nNOS and dystrophin immunostaining images of the gastrocnemius muscle, the nNOS signal co-localized with dystrophin was extracted as a membrane nNOS by image

processing. The right side of the section was the deep region of the muscle. Fluorescence intensity is indicated by pseudo-colors. In the upper right corner, the pseudo-color conversion lookup table is shown. Inserts show magnified views of each image. (**b**) Frequency distribution of gray values for membrane nNOS immunostaining of the WT-Tg gastrocnemius muscle sections. Compared to WT-Tg CON (control) samples, the gray value distribution shifted to lower values in the WT-Tg DEN (denervated) samples. *: a significant difference was observed between CON and DEN. (**c**) Frequency distribution of gray values for membrane nNOS immunostaining of the *Hspg2*$^{-/-}$-Tg gastrocnemius muscle sections. A smaller shift in the frequency distribution was observed compared to *Hspg2*$^{-/-}$-Tg CON and DEN. (**d**) Immunofluorescence images of gastrocnemius muscle whole cross-sections showing dystrophin expression. (**e**) Frequency distribution of gray values for dystrophin immunostaining of the WT-Tg gastrocnemius muscle sections. Gray value distribution did not change significantly between WT-Tg CON and WT-Tg DEN ($P > 0.05$). (**f**) Frequency distribution of gray values for dystrophin immunostaining of the *Hspg2*$^{-/-}$-Tg gastrocnemius muscle sections. Comparison of *Hspg2*$^{-/-}$-Tg CON and DEN revealed significant differences at lower gray values ($P < 0.05$), but the overall shift was slight. (**g**) Percent change (from CON to DEN) in the mean gray value of dystrophin. A significant difference was not observed between WT-Tg CON and DEN nor between *Hspg2*$^{-/-}$-Tg CON and DEN ($P > 0.05$). Values are expressed as the mean ± SD. (h) The corrected nNOS gray value calculated from the membrane nNOS gray value and dystrophin immunostaining gray value. †: a significant difference was observed between WT-Tg CON and WT-Tg DEN ($P < 0.05$). *: a significant difference was observed between WT-Tg CON and *Hspg2*$^{-/-}$-Tg CON ($P < 0.05$).
